# Co-expression of fibroblast growth factor receptor 3 with mutant p53, and its association with worse outcome in oropharyngeal squamous cell carcinoma

**DOI:** 10.1371/journal.pone.0247498

**Published:** 2021-02-24

**Authors:** Sreenivas Nannapaneni, Christopher C. Griffith, Kelly R. Magliocca, Wanqi Chen, Xueying Lyu, Zhengjia Chen, Dongsheng Wang, Xu Wang, Dong M. Shin, Zhuo G. Chen, Nabil F. Saba

**Affiliations:** 1 Department of Hematology and Medical Oncology, Winship Cancer Institute of Emory University School of Medicine, Atlanta, Georgia, United States of America; 2 Department of Pathology, Cleveland Clinic, Cleveland, Ohio, United States of America; 3 Department of Pathology & Laboratory Medicine, Emory University School of Medicine, Atlanta, Georgia, United States of America; 4 Department of Biostatistics and Bioinformatics, Emory University School of Public Health, Atlanta, Georgia, United States of America; 5 Department of Epidemiology & Biostatistics, University of Illinois Cancer Center, Chicago, Illinois, United States of America; Centro Nacional de Investigaciones Oncologicas, SPAIN

## Abstract

Fibroblast growth factor receptor 3 (FGFR3) is expressed in squamous cell carcinoma of the head and neck (SCCHN) including oropharyngeal squamous cell carcinoma (OPSCC) and is a potential therapeutic target. However, information on its correlation with other relevant cancer related proteins stratified by p16 status and its prognostic significance in OPSCC is limited. We examined FGFR3 expression and its correlation with clinical characteristics, p16 status, and mutant p53 (mp53) among 220 retrospectively collected OPSCC cases and 40 prospectively collected SCCHN cases, including a majority of OPSCC. Correlations of FGFR3 Weighted Index (WI) with p16 status and mp53 WI as well as its association with disease-free survival (DFS) and overall survival (OS) were evaluated. FGFR3 expression was detected in 61% and 70% of cases in cohorts 1 and 2, respectively. FGFR3 level was significantly higher in p16-negative tumors in both cohorts (p<0.001 and 0.006). FGFR3 expression was highly correlated with mp53 expression in both p16 + and p16– OPSCC (p<0.0001 and p = 0.0006, respectively). In cohort 1, univariate analysis showed that FGFR3 was associated with DFS but not OS. Kaplan-Meier analysis showed that higher FGFR3 and mp53 level correlated with worse DFS (p = 0.025) and OS (p = 0.009). As expected, p16 positive status was associated with improved OS and DFS (p<0.001 for both). Our results suggest that high FGFR3 expression is associated with p16 negative status and mp53 expression in OPSCC and correlates with a worse clinical outcome. The biological relationship between FGFR3 and mp53 in OPSCC deserves further investigation.

## Introduction

According to the American Cancer Society, the mortality rate for squamous cell carcinoma of the head and neck (SCCHN) is decreasing, however, there has been a rise in oropharyngeal squamous cell carcinoma (OPSCC) due to human papilloma virus (HPV) infection [1, 2]. Approximately 65,410 people were estimated to be diagnosed with SCCHN and 14,620 to die of this disease in 2019 [3] HPV-positive SCCHN occurs most frequently in the oropharynx (tonsil and base of tongue), whereas HPV-negative SCCHN occurs in all head and neck sites. HPV-positive SCCHN has better responses to radiation/chemotherapy as well as significantly improved outcomes compared to HPV-negative SCCHN, which usually requires more aggressive treatment [2, 4, 5]. It is also notable that mutations in one of the most common tumor suppressor proteins, p53, contribute to the development of SCCHN, and wild type p53 has a low expression in HPV-positive tumors [6].

Fibroblast growth factor receptor (FGFR), a receptor tyrosine kinase (RTK), consists of 4 family members, FGFR1, 2, 3, and 4. The FGFRs regulate important biological processes including cell proliferation and differentiation as well as tissue repair [7, 8]. FGFR3 downstream signaling pathways include PI3K/AKT and RAS-MEK-ERK, which are common signaling axes that support cancer cell progression [9]. Increased expression of FGFR3 has been observed in various cancer types including sarcoma, multiple myeloma, breast, prostate, lung, brain, and head and neck cancers [7, 10–13]. However, there is no clear evidence that this expression implicates FGFR3 as a possible underlying molecular driver of these cancers.

Mutations in *FGFR3*, including amplification and the *FGFR3-TACC3* fusion gene [14, 15], have been identified. In SCCHN, the *FGFR3-TACC3* fusion was identified and *FGFR* mutations were studied mainly in HPV-positive cases [6, 16, 17], suggesting this RTK may serve as a target for treatment in this specific SCCHN population. However, although FGFR inhibitors, such as small molecules and antibodies, have shown some clinical benefits in early phase trials, the clinical response rate to these agents is relatively low despite FGFR alterations [7]. Furthermore, some patients without *FGFR* alteration have also responded to these inhibitors [7], implicating other possible pathways besides FGFR in predicting clinical response to these agents.

To understand the potential role of FGFR3 in both HPV-positive and -negative SCCHN, we examined FGFR3 protein expression in two cohorts of SCCHN patients consisting mostly of OPSCC and examined the correlation with mutant p53 (mp53) and HPV status using p16 as a surrogate biomarker for HPV. The potential prognostic value of FGFR3 was evaluated.

## Materials and methods

### Patient characteristics

This study was conducted using retrospectively and prospectively collected formalin-fixed paraffin-embedded (FFPE) specimens from two cohorts of patients. A retrospective cohort of 220 patients with a diagnosis of OPSCC (**S1 Table**) and 40 prospectively collected tissues from patients with SCCHN, including 29 OPSCC and 11 oral cavity SCC (**S2 Table**) were analyzed. All specimens were obtained under an Institutional Review Board (IRB)-approved protocol at Emory University and patient information collected in compliance with the Health Insurance Portability and Accountability Act (HIPAA). Patient specimens were obtained at diagnosis, prior to any treatment between the years 1994–2016. Key clinical parameters including gender, race, smoking status (never, former, and current), and pathologic data (differentiation, tumor stage, and presence of lymph node metastasis) were considered variables in this analysis. Data was obtained from clinically annotated source documents including pathologic reports and other relevant electronic medical records.

### Immunohistochemistry (IHC)

FFPE tumor tissue sections were pre-heated at 60°C for 30 minutes and washed through a series of xylene and alcohol containers followed by antigen retrieval using 1X citrate buffer for 10 minutes in a microwave. The slides were cooled for 30 minutes at room temperature and quenched using 3% hydrogen peroxide in distilled water. The sections were washed and then blocked using 2.5% normal horse serum following the manufacturer’s instructions (Vectastain Kit, Vector Laboratories, Burlingame, CA). Besides hematoxylin and eosin (H&E) staining, standard immunohistochemistry (IHC) was used to stain for p16 (1:100, Delta Biolab, Gilroy, CA), FGFR3 (PA2143, Boster Bio, Pleasanton, CA.) and mp53 (AB32049, Abcam, Cambridge, MA.) using primary antibodies followed by corresponding secondary antibodies, visualized by DAB and counterstained with hematoxylin. Positive signals were counted in five random fields under ×100 magnification and were quantified using weighted index method. WI = Intensity × % of positive staining. The intensity of staining was scored as negative (0), weak (1+), intermediate (2+), and strong (3+), respectively. p16 positivity was determined if > 70% of tumor cells stained with p16 specific antibody in the nuclei and cytoplasm [18, 19]. These quantifications were determined by at least 2 individuals blindly and independently. In the case of disagreement, two pathologists had to come to a consensual agreement.

### Statistical analysis

Descriptive statistics was used to summarize the characteristics for each patient. Frequency and percentage for categorical variables were presented. For numeric variables, mean, median, and standard deviation were calculated. In the univariate analysis with FGFR3, the ANOVA test was used to compare FGFR3 between different categories. Pearson correlation coefficient was calculated to examine the relationship between FGFR3 with continuous variables. Stratified analyses were conducted to assess the confounding effect of p16 on the relationship between FGFR3 and mp53, cytoplasmic mp53, and nuclear mp53 after adjusting for p16, respectively. The general linear model (GLM) was further employed in multivariate analyses to assess the adjusted relationship between FGFR3 and mp53, cytoplasmic mp53, nuclear mp53 after adjusting for p16, respectively. The univariate association of each covariate with overall survival (OS) or disease free survival (DFS) was assessed using the Cox proportional hazards model. Also, Kaplan-Meier (K-M) plot and log-rank test were presented. A multivariate model was fitted by backward selection method with removal criteria alpha = 0.20. All statistical analyses were performed using SAS 9.4 (SAS Institute, Inc., Cary, North Carolina).

## Results

### FGFR3 and mp53 expression in OPSCC

**Fig 1:**
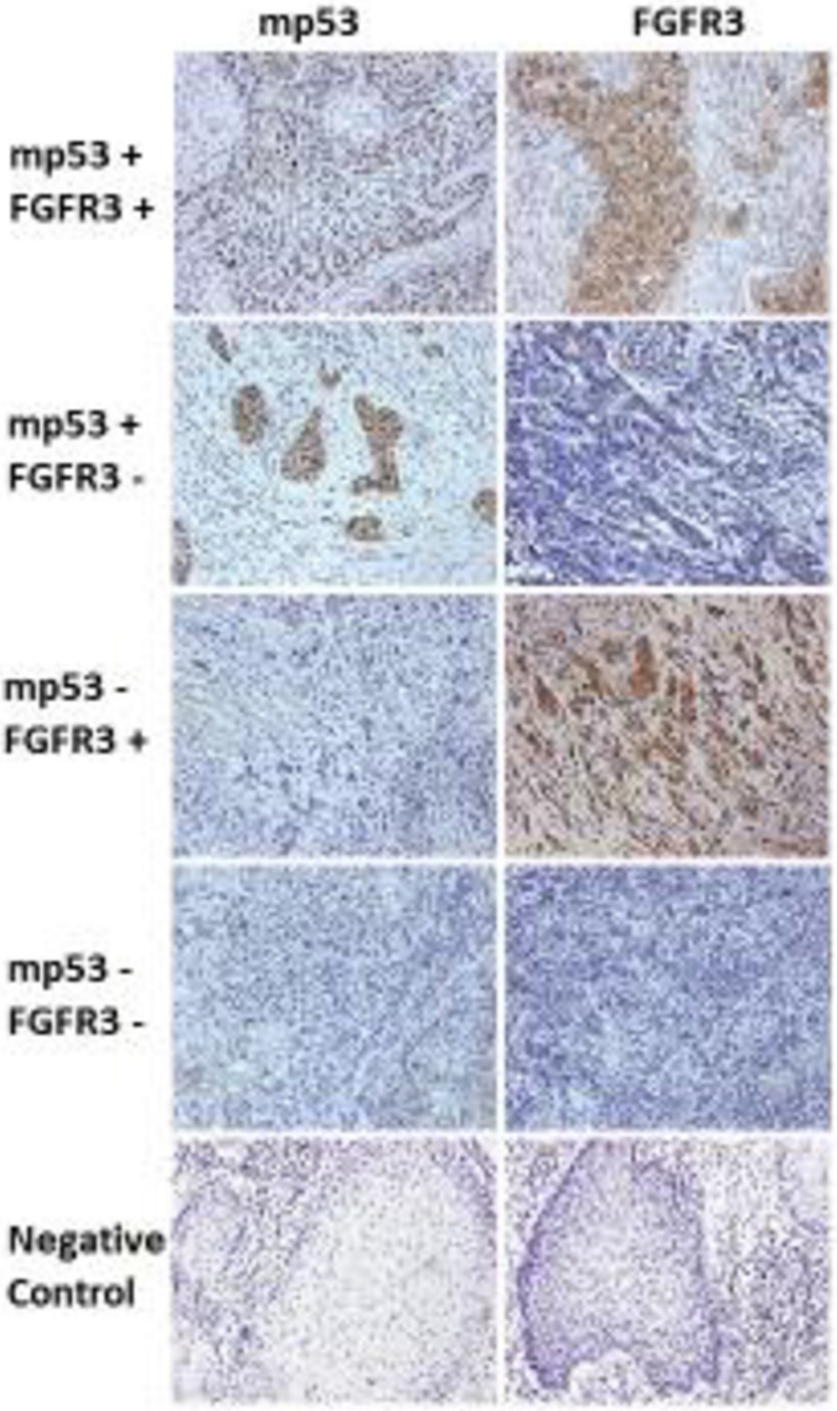
mp53 and FGFR3 staining in samples from patients with SCCHN. IHC staining of mp53 in SCCHN samples shows mainly nuclear and some cytoplasmic expression patterns. IHC staining of FGFR3 shows membrane and cytoplasmic expression. Four representative images are presented: mp53+/FGFR3+, mp53+/FGFR3-, mp53-/FGFR3+, mp53-/FGFR3- and negative control. (200 x magnification).

Among the first cohort of 220 OPSCC cases, 62.2% (135/220) of cases were p16 positive, 78% (91/116 available tissues) were positive for mp53 expression, and 61% (134/220) were positive for FGFR3. In the second cohort, 86% (25/29 OPSCC) of cases were p16 positive, 88% (35/40) expressed mp53, and 70% (30/40) were positive for FGFR3. A higher percentage of cases expressed mp53 and FGFR3 in the second cohort, which may reflect the fact that there were 11 cases of non-OPSCC, all of which expressed mp53 (**Fig 1**). The negative control slide had no primary antibody in the incubation buffer. The specificity of FGFR3 antibody was supported by both positive and negative controls (**S1 Fig**). Membrane, cytoplasmic, and nuclear localizations of FGFR3 are consistent with the reported literature [20, 21]. Expression levels of FGFR3 and total, cytoplasmic, and nuclear mp53 are summarized in **S3** and **S4 Tables**.

### Correlation of FGFR3 expression with p16 status and mp53

FGFR3 level was significantly higher in p16-negative tumors in both cohorts (p<0.001 and 0.006) (**Tables 1** and **2**). Furthermore, FGFR3 expression was highly associated with total, cytoplasmic, and nuclear mp53 in cohort 1 (**Table 3**), with cytoplasmic mp53 being more significantly correlated (p < 0.001) than nuclear mp53 (p = 0.050). Further stratifying by p16 status, we observed that in both p16 positive and negative tumors, FGFR3 correlated with cytoplasmic mp53, but not nuclear mp53 (p = 0.0009 *vs*. 0.517 and 0.0007 *vs*. 0.319, respectively, **Tables 4** and **5**). In cohort 2, FGFR3 was not found to be significantly correlated with mp53 (**S5 Table**), which may be a result of the smaller sample size. Pearson correlation consistently showed that in both cohorts, mp53 and FGFR3 were inversely associated with p16 (**S6** and **S7 Tables**), suggesting that FGFR3 and mp53 association is predominantly seen in p16-negative SCCHN.

**Table 1:** Univariate association of FGFR3 with clinical characteristics in cohort 1.

FGFR3				
Variable	Level	N	Mean	ANOVA p-Value
Gender	Male	168	61.90	
	Female	52	55.67	0.563
Smoking	Never	40	38.88	
	Former	85	54.35	
	Current	84	77.50	**0.006**
	Missing	11		
p16	Positive	135	46.11	
	Negative	82	84.76	**< 0.001**
	Missing	3		
Grade	MD	74	94.66	
	NK	120	41.08	
	PD	5	46.00	
	WD	17	66.47	**< 0.001**
	Missing	4		
T-Stage	1	71	49.51	
	2	81	74.88	
	3	16	67.50	
	4	33	50.00	0.079
	Missing	19		
Node Status	0	42	67.62	
	1	28	64.64	
	2	119	59.75	
	3	14	72.14	0.869
	Missing	17		
Stage	I	14	55.00	
	II	30	82.50	
	III	19	53.16	
	IV	144	59.79	0.588
	Missing	13		

**Table 2:** FGFR3 association with clinical characteristics in cohort 2.

FGFR3				
Variable	Level	N	Mean	ANOVA p-Value
Gender	Male	31	78.46	
	Female	9	164.44	**0.002**
Smoking	Never	15	65.36	
	Former	16	111.07	
	Current	8	148.33	**0.049**
	Missing	1		
p16	Positive	25	73	
	Negative	6	115	
	Unknown	6	166.67	**0.006**
	Missing	3		
Grade	MD	17	139.12	
	NK	16	67.92	
	PD	6	66	
	WD	1	10	**< 0.016**
T-Stage	1	5	72.50	
	2	13	73.75	
	3	2	20.00	
	4	20	135.59	**0.032**
Node Status	0	8	97.14	
	1	1	100	
	2	30	101.48	
	3	1	103.21	0.991
Stage	I	1	20.00	
	II	3	86.67	
	IV	36	104.52	0.517

**Table 3:** Correlation of FGFR3 with p53 and mp53 in cohort 1.

		FGFR3	
Variable	N	Pearson CC	Pearson p-Value
mp53	166	0.513	< 0.001
Cytoplasmic mp53	166	0.469	< 0.001
Nuclear mp53	166	0.152	0.050

**Table 4:** Pearson correlation coefficients (p-values) between FGFR3 and mp53, cytoplasmic and nuclear mp53 when p16 is negative.

	mp53	Cytoplasmic mp53	Nuclear mp53
**FGFR3**	0.48475	0.43300	0.08850
	(0.0002)	(0.0009)	(0.5166)

**Table 5:** Pearson correlation coefficients (p-values) between FGFR3 and mp53, cytoplasmic and nuclear mp53 when p16 is positive.

	mp53	Cytoplasmic mp53	Nuclear mp53
**FGFR3**	0.33522	0.33158	0.10009
	(0.0006)	(0.0007)	(0.3193)

### Univariate and multivariate analyses of FGFR3 associated with clinical characteristics, OS, and DFS

In addition to p16 status, FGFR3 expression was highly associated with grade and smoking history in cohort 1 (**Table 1**). In cohort 2, FGFR3 was highly associated with smoking history and T-stage (**Table 2**).

In cohort 1, univariate analysis showed that FGFR3 was associated with DFS but not OS (**Tables 6** and **7**). In addition, p16 status, smoking, tumor stage, and mp53 were found to correlate with both OS and DFS. Kaplan-Meier analysis of the first cohort of patients (OPSCC) also showed that FGFR3 expression is associated DFS, not OS (**S2 Fig**). The factors used in the univariate analysis were further considered in the multivariate analysis which also showed that FGFR3 expression was correlated with DFS (p = 0.005), but not OS (p = 0.172) **(Table 8).** Negative p16 status, current smoking, and T-stage 3 and 4 were associated with worse OS (**Table 9**). The mp53 and T-stage 3 and 4 were associated with worse DFS (**Table 8**).

**Table 6:** Cohort 1, univariate analysis of overall survival.

			OS	
Covariate	Level	N	Hazard Ratio	Log Rank p-Value
Gender	Male	152	0.69 (0.43–1.10)	0.115
	Female	49	-	
p16	Positive	117	3.43 (2.22–5.32)	**< 0.001**
	Negative	81	-	
Smoking	0	38	0.13 (0.05–0.32)	**< 0.001**
	1	78	0.41 (0.26–0.66)	
	2	80	-	
Grade	MD	51	0.66 (0.26–1.54)	0.273
	NK	82	0.46 (0.20–0.105)	
	PD	4	0.77 (0.16–3.74)	
	WD	13	-	
T Stage	1	70	0.29 (0.15–0.55)	**< 0.001**
	2	72	0.41 (0.23–0.73)	
	3	14	1.09 (0.49–2.43)	
	4	32	-	
N Stage	0	40	1.73 (0.60–5.01)	0.545
	1	25	1.06 (0.33–3.46)	
	2	24	1.47 (0.48–4.48)	
	3	14	-	
Stage	I	14	1.08 (0.46–2.51)	0.763
	II	25	1.00 (0.55–1.81)	
	III	16	0.62 (0.25–1.55)	
	IV	134	-	
FGFR3	< Median	99	0.78 (0.51–1.20)	0.259
	> = Median	102	-	
mp53	0	113	0.56 (0.36–0.86)	**0.007**
	1	88	-	
Nuclear mp53	0	153	0.93 (0.57–1.50)	0.754
	1	48	-	
Cytoplasmic mp53	0	104	0.94 (0.62–1.44)	0.790
	1	97	-	

**Table 7:** Cohort 1, univariate analysis of disease free survival.

			DFS	
Covariate	Level	N	Hazard Ratio	Log Rank p-Value
Gender	Male	161	0.42 (0.22–0.79)	**0.005**
	Female	50	-	
p16	0	79	3.53 (1.90–6.55)	**< 0.001**
	1	129	-	
Smoking	0	39	0.41 (0.17–0.98)	**< 0.001**
	1	80	0.47 (0.24–0.93)	
	2	82	-	
Grade	MD	48	0.83 (523033.5 (0.00-)	0.933
	NK	80	423857.6 (0.00-)	
	PD	4	590902.1 (0.00-)	
	WD	16	481483.0 (0.00-)	
T Stage	1	71	0.33 (0.13–0.80)	**0.009**
	2	79	0.47 (0.21–1.07)	
	3	15	1.31 (0.46–3.70)	
	4	29	-	
N Stage	0	42	7426036 (0.00-)	**0.031**
	1	27	2194343 (0.00-)	
	2	24	4271671 (0.00-)	
	3	14	-	
Stage	I	14	2.81 (1.08–7.32)	0.145
	II	28	1.10 (0.46–2.68)	
	III	19	0.78 (0.24–2.58)	
	IV	133	-	
FGFR3	< Median	104	0.50 (0.27–0.94)	**0.029**
	> = Median	107	-	
mp53	0	122	0.47 (0.26–0.87)	**0.014**
	1	89	-	
Nuclear mp53	0	163	0.81 (0.41–1.58)	0.529
	1	48	-	
Cytoplasmic mp53	0	112	1.16 (0.63–2.12)	0.641
	1	99	-	

**Table 8:** Multivariate survival analysis on disease free survival.

		DFS Months		
		----------------------------------------		
Covariate	Level	Hazard Ratio	HR P-value	Type3 P-value
FGFR3	> = Median	2.75 (1.35–5.62)	**0.005**	**0.005**
	<Median	-	-	
mp53	> = Median	2.09 (1.00–4.36)	**0.049**	**0.049**
	<Median	-	-	
T-stage	4	3.09 (1.22–7.85)	**0.017**	**0.010**
	3	3.84 (1.29–11.48)	**0.016**	
	2	1.04 (0.44–2.45)	0.932	
	1	-	-	

* Number of observations in the original data set = 220. Number of observations used = 170.

** Backward selection with an alpha level of removal of .20 was used.

**Table 9:** Multivariate survival analysis on overall survival.

		OS Months		
		----------------------------------------		
Covariate	Level	Hazard Ratio	HR P-value	Type3 P-value
p16	Negative	2.78 (1.68–4.60)	**< .001**	**< .001**
	Positive	-	-	
Smoking	Current	2.60 (1.11–6.09)	**0.027**	**0.048**
	Former	1.65 (0.70–3.88)	0.255	
	Never	-	-	
T-stage	4	2.84 (1.47–5.47)	**0.002**	**0.001**
	3	2.63 (1.14–6.04)	**0.023**	
	2	1.03 (0.57–1.86)	0.923	
	1	-	-	

* Number of observations in the original data set = 220. Number of observations used = 179.

** Backward selection with an alpha level removal of .20 was used.

**Fig 2:**
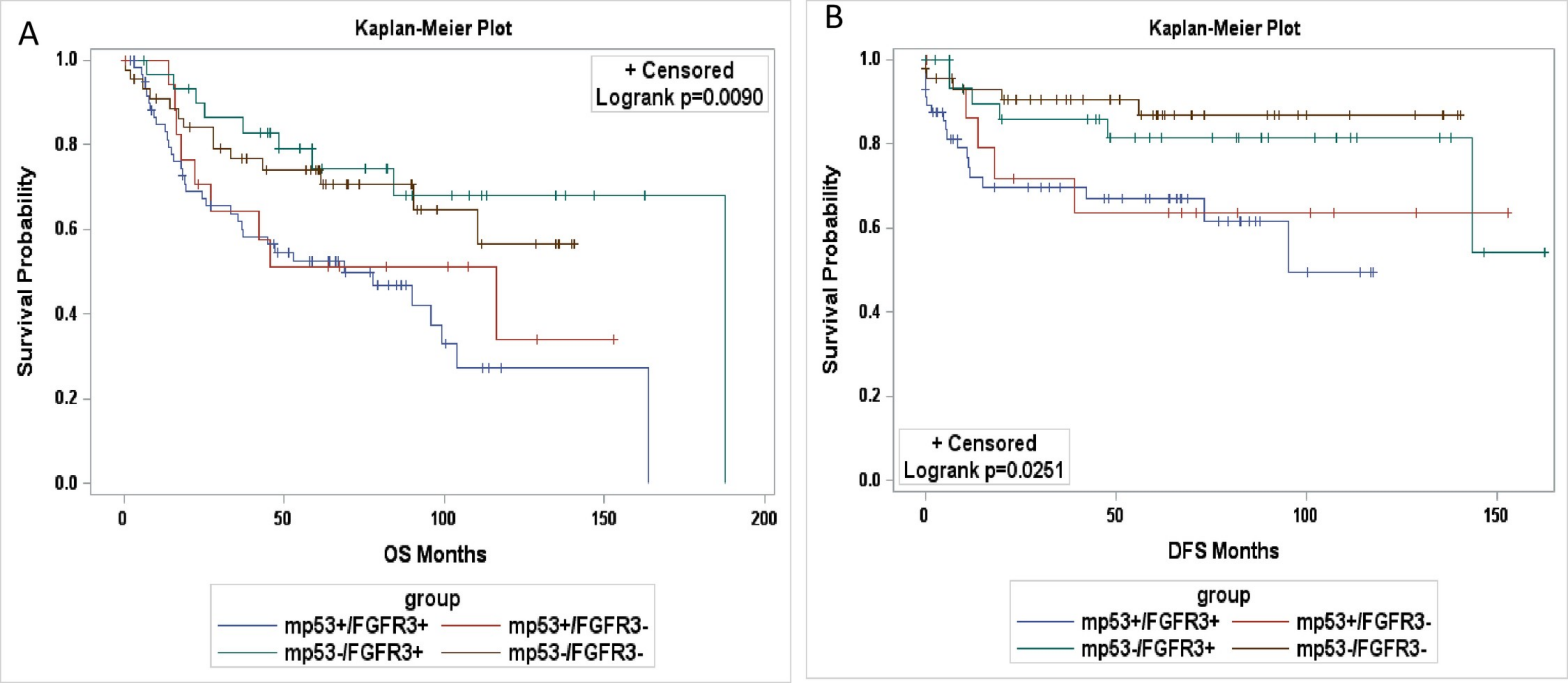
Kaplan–Meier (KM) estimates of overall survival (OS) and disease-free survival (DFS) using FGFR3 and mp53 levels in patients with OPSCC. KM plots were generated using median weighted index (WI) of mp53 (3.5) and FGFR3 (35) as the cut off values (>median as positive and ≤ median as negative). High FGFR3 and mp53 levels correlated with worse OS (A) and DFS (B).

Furthermore, Kaplan-Meier analysis illustrated that mp53 and FGFR3 were significantly associated with worse OS and DFS using medians of both FGFR3 and mp53 as the cut-off values (*p* = 0.009 and 0.025, respectively) (**Fig 2**).

No correlation with either OS or DFS was identified in the second cohort of samples, due to either the small sample size or short follow up time. Therefore, the results were mainly from the first cohort.

## Discussion

SCCHN has been classified as an HPV-driven or non–HPV-driven disease. However, a reliable molecularly based classification that could impact clinical decision and the use of molecularly targeted agents is lacking in this disease. Despite the fact that FGFR remains one of the common genetic alterations in SCCHN, few studies have evaluated or targeted this pathway in SCCHN and none has produced data convincing enough to be practice changing. Although FGFR3 has been studied in SCCHN [10, 22, 23], its expression level has not been carefully correlated with common alterations in SCCHN such as HPV and p53 mutational status. In addition, there is limited and conflicting data on its prognostic significance in SCCHN [13]. Our findings are the first to confirm a strong correlation between FGFR3 and p16 negative status in SCCHN, as well as an inverse correlation between both mp53 and FGFR3 with p16 status. These findings suggest a more significant role for FGFR3 in p16-negativeSCCHN. It is of interest that FGFR3 and mp53 were both associated with a worse clinical outcome in addition to being more commonly co-expressed, raising the question of potential biological interactions between FGFR3 and mp53 and opening the door for studies to elucidate possible mechanistic interactions. A recent publication suggested that p53 IHC could be a surrogate marker for p53 mutation [24]. It is known that wild-type p53 is not stable and the previous IHC staining for p53 most likely reflected expression of mp53. In our study, we used an antibody which recognizes mp53 only. We found no nuclear expression of mp53 in some tissues using this specific antibody, but we cannot rule out whether there is or not the wild-type p53 which is not stable in tumor tissues. Other limitations in our analysis included its focus on expression levels of FGFR3 and its correlation with mp53 only but did not correlate FGFR3 mRNA and the active FGFR3. Despite this limitation, our findings raise the possibility of an improved molecular classification of mp53-altered SCCHN, particularly OPSCC, based on these findings, in addition to possibly enhancing patient selection for future clinical trials in HPV-unrelated disease.

Of further interest, and consistent with TCGA data, the FGFR3-TACC3 fusion gene was identified in two cases of HPV-positive SCCHN in our prospective second cohort [25]. This suggests that in HPV-positive SCCHN, activation of FGFR3 may be driven by genomic alterations that are different from those in HPV-negative disease. Recent studies have examined *FGFR* gene mutation and amplification in HPV-positive SCCHN and described the occurrence of FGFR3 [16, 17]; TCGA reported two cases of *FGFR3-TACC3* fusion gene only in HPV-positive tumors [6]. The *FGFR3-TACC3* fusion gene was first identified in glioblastoma multiforme (GBM) by two independent groups [14, 15, 26, 27]. Both reported that the *FGFR3-TACC3* cDNA contains an open reading frame coding for a protein with 1048 amino acids. This fusion protein includes the FGFR3 N terminus (residues 1–758) and the TACC3 C terminus (residues 549–838). FGFR3 is a receptor tyrosine kinase, while TACC3 (transforming acidic coiled-coil) is a centrosomal protein involved in mitosis [28, 29]. In addition to brain tumors, to date, the FGFR3-TACC3 fusion protein has been reported in several solid tumors [30], including non-small cell lung cancer [31], cervical cancer [32], esophageal cancer [33], gastric cancer [34], and SCCHN [35].

In HPV-unrelated disease, our findings suggest a proportional correlation between FGFR3 and mp53 expressions. A regulatory role of mp53 on FGFR3 expression may possibly exist, however needs to be confirmed in further investigation. In SCCHN, over 70% of p53 are mutated in SCCHN [6]. Though p53 is a tumor suppressor, mp53 may work as an oncogenic transcription activator [36–38]. FGFR3 and p53 play different roles in cancer development. FGFR3 initiates multiple signaling pathways to support cell proliferation, while mutation or loss of p53 function results in loss of cell cycle regulation leading to tumor progression. Two publications using mouse models of carcinogenesis reported that the *FGFR3-TACC3* fusion gene could effectively induce spontaneous brain or lung tumors in mice only with p53 knockout, suggesting that abnormal FGFR3 activation in the absence of normal p53 function may enhance cancer cell growth [15, 39]. Our findings that simultaneous FGFR3 and mp53 alteration is correlated with worse DFS and OS support this hypothesis and urge the need for confirmatory studies. If such a correlation is confirmed, this could have implications for patient stratification and the identification of a unique subgroup of HPV-negative SCCHN. Since mp53 may function as an oncoprotein, a possible regulatory effect on FGFR3 expression deserves further investigation.

In the current study, we used p16 as a reliable surrogate biomarker for HPV-associated OPSCC since in this disease, there is functional inactivation of RB by the viral oncoprotein E7, which is known to cause upregulation of p16 expression. In other SCCHN sub-populations, such as oral cavity and larynx, p16 is not an indicator for HPV-related cancer. However, though our second independent cohort contained 11 non-OPSCC samples, the correlation observed between FGFR3 and p16 was consistent with that in the first cohort.

Currently, several FGFR inhibitors are under clinical investigation [7]. All of these agents target pan-FGFR or multiple RTKs. Two studies using dovitinib, a multi-RTK inhibitor, selected overexpressed or mutated FGFR3 urothelial malignancies [40, 41]. In SCCHN, the clinical applications of FGFR-based therapy have focused on metastatic and/or recurrent disease and used pan-FGFR inhibitors (ClinicalTrial.gov identifier: NCT02152254 and NCI1976741, respectively) [42–44]. These trials included multiple tumor types and suggested that FGFR overexpressing cancers through FGFR mRNA levels tended to benefit from small molecule inhibitors of FGFR1-4 such as rogaratinib. The head and neck cancer cohorts within these studies consisted, however, of very few patients rendering any firm conclusions difficult to draw. Though our study did not look specifically for aberrant expressions of mutant p53 and FGFR3 mainly in OPSCC as compared with normal tissue, which is a limitation of this study, our study suggests that FGFR3 may play a more significant role in HPV-negative OPSCC along with mp53 alteration. Our findings, if further confirmed, open the door for a potentially improved biomarker-based selection and could inform the design of future clinical trials in OPSCC cases particularly.

## Supporting information

S1 Fig Controls of FGFR3 staining used in the manuscript (PA2143, Boster Bio, Pleasanton, CA.). Mouse lung tissue—negative control (A) and positive staining (B and C); FGFR3 positive OPSCC tissue (D). Magnification 200X.
(PPTX)

S2 Fig Kaplan-Meier plot of FGRF3 expression in OPSCC. The cut-off is the median expression values as indicated. A) Overall survival. B) Disease free survival (DFS) Kaplan Meier plot shows shorter DFS time for patients with high FGFR3 expression.
(PPTX)

S1 Table Cohort 1 patient characteristics. (DOCX)

S2 Table Cohort 2 patient characteristics. (DOCX)

S3 Table Expression levels of FGFR3 and mp53 in cohort 1. (DOCX)

S4 Table Expression levels of FGFR3 and mp53 in cohort 2. (DOCX)

S5 Table Correlation of FGFR3 with mP53 in cohort 2. (DOCX)

S6 Table Cohort 1 Pearson correlation coefficient (p-values) between p16 and FGFR3, mp53, cytoplasmic and nuclear mp53. (DOCX)

S7 Table Cohort 2 Pearson correlation coefficient (p-values) between p16 and FGFR3, mp53, cytoplasmic and nuclear mp53. (DOCX)
