# Supplementary material for: Co-expression of fibroblast growth factor receptor 3 with mutant p53, and its association with worse outcome in oropharyngeal squamous cell carcinoma
Source: PLoS One. 2021 Feb 24;16(2):e0247498. doi: 10.1371/journal.pone.0247498 (PMC7904228; doi:10.1371/journal.pone.0247498)
Supplement: S1 Fig — Mouse lung tissue—negative control (A) and positive staining (B and C); FGFR3 positive OPSCC tissue (D). Magnification 200X. (PPTX) [file pone.0247498.s001.pptx]

## Slide 1
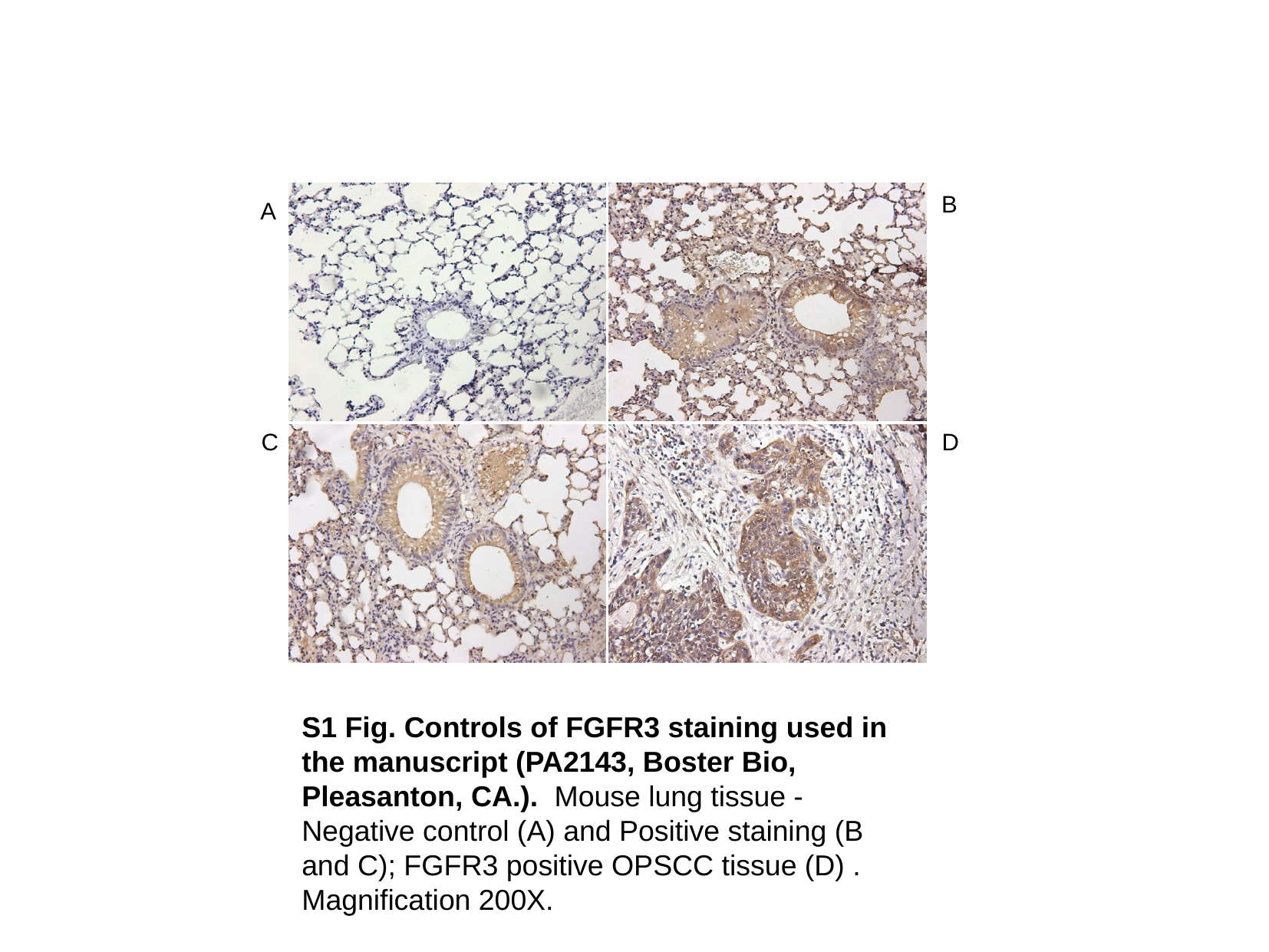

B
A
C
D
S1 Fig. Controls of FGFR3 staining used in the manuscript (PA2143, Boster Bio, Pleasanton, CA.). Mouse lung tissue - Negative control (A) and Positive staining (B and C); FGFR3 positive OPSCC tissue (D) . Magnification 200X.
