# Supplementary material for: Co-expression of fibroblast growth factor receptor 3 with mutant p53, and its association with worse outcome in oropharyngeal squamous cell carcinoma
Source: PLoS One. 2021 Feb 24;16(2):e0247498. doi: 10.1371/journal.pone.0247498 (PMC7904228; doi:10.1371/journal.pone.0247498)
Supplement: S2 Fig — The cut-off is the median expression values as indicated. A) Overall survival. B) Disease free survival (DFS) Kaplan Meier plot shows shorter DFS time for patients with high FGFR3 expression. (PPTX) [file pone.0247498.s002.pptx]

## Slide 1
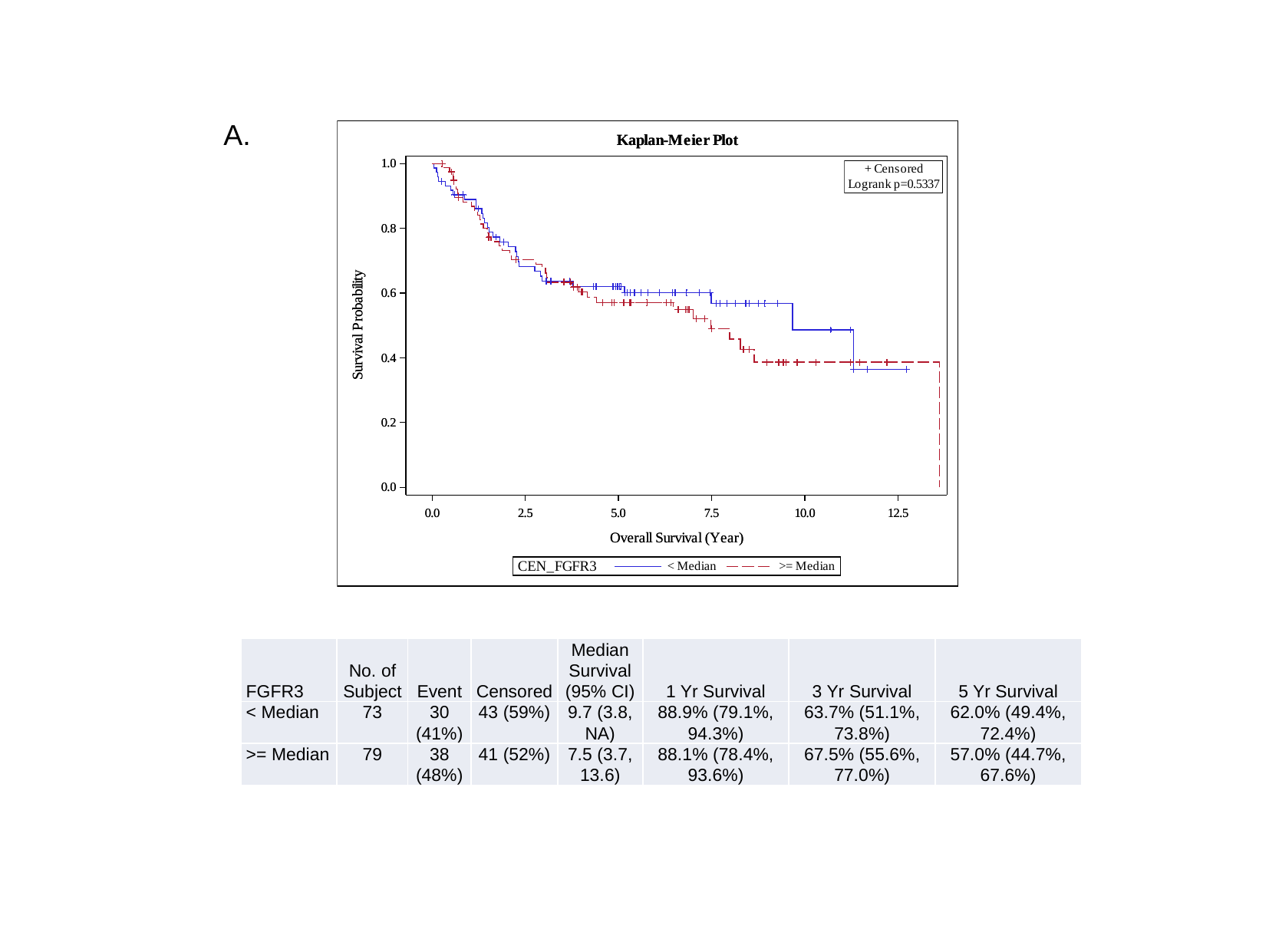

A.
| FGFR3 | No. of Subject | Event | Censored | Median Survival (95% CI) | 1 Yr Survival | 3 Yr Survival | 5 Yr Survival |
| --- | --- | --- | --- | --- | --- | --- | --- |
| < Median | 73 | 30 (41%) | 43 (59%) | 9.7 (3.8, NA) | 88.9% (79.1%, 94.3%) | 63.7% (51.1%, 73.8%) | 62.0% (49.4%, 72.4%) |
| >= Median | 79 | 38 (48%) | 41 (52%) | 7.5 (3.7, 13.6) | 88.1% (78.4%, 93.6%) | 67.5% (55.6%, 77.0%) | 57.0% (44.7%, 67.6%) |

## Slide 2
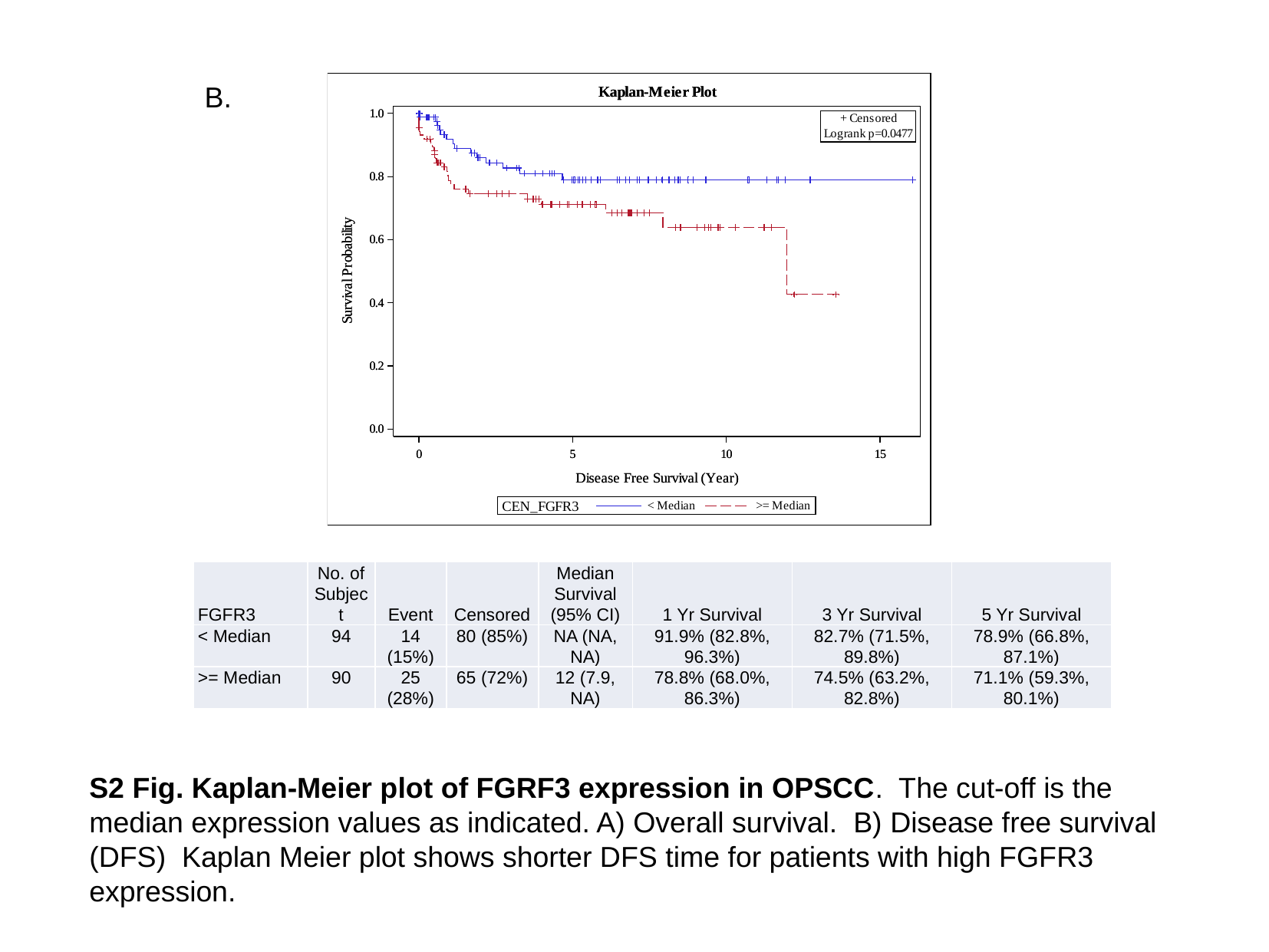

B.
| FGFR3 | No. of Subject | Event | Censored | Median Survival (95% CI) | 1 Yr Survival | 3 Yr Survival | 5 Yr Survival |
| --- | --- | --- | --- | --- | --- | --- | --- |
| < Median | 94 | 14 (15%) | 80 (85%) | NA (NA, NA) | 91.9% (82.8%, 96.3%) | 82.7% (71.5%, 89.8%) | 78.9% (66.8%, 87.1%) |
| >= Median | 90 | 25 (28%) | 65 (72%) | 12 (7.9, NA) | 78.8% (68.0%, 86.3%) | 74.5% (63.2%, 82.8%) | 71.1% (59.3%, 80.1%) |
S2 Fig. Kaplan-Meier plot of FGRF3 expression in OPSCC. The cut-off is the median expression values as indicated. A) Overall survival. B) Disease free survival (DFS) Kaplan Meier plot shows shorter DFS time for patients with high FGFR3 expression.
