# Supplementary material for: Co-expression of fibroblast growth factor receptor 3 with mutant p53, and its association with worse outcome in oropharyngeal squamous cell carcinoma
Source: PLoS One. 2021 Feb 24;16(2):e0247498. doi: 10.1371/journal.pone.0247498 (PMC7904228; doi:10.1371/journal.pone.0247498)
Supplement: S1 Table — (DOCX) [file pone.0247498.s003.docx]

S1 Table. Cohort 1 Patient Characteristics

| Variable | Level | N (%) = 220 |
| --- | --- | --- |
| Gender  Smoking  p16  Grade  T – Stage  Node Status  Stage | Male  Female  Never  Former  Current  Missing  Positive  Negative  Missing  MD  NK  PD  WD  missing  1  2  3  4  Missing  0  1  2  3  Missing  I  II  III  IV  Missing | 168 (76.4)  52 (23.6)  40 (19.1)  85 (40.7)  84 (40.2)  11  135 (62.2)  82 (37.8)  3  74 (33.6)  120 (54.5)  5 (2.3)  17 (7.7)  4 (01.8)  71 (35.3)  81 (40.3)  16 (8.0)  33 (16.4)  19  42 (38.5)  28 (25.7)  119 (58.6)  14 (12.8)  17  14 (7.0)  30 (14.5)  19 (9.5)  144 (69.6)  13 |
