# Supplementary material for: Co-expression of fibroblast growth factor receptor 3 with mutant p53, and its association with worse outcome in oropharyngeal squamous cell carcinoma
Source: PLoS One. 2021 Feb 24;16(2):e0247498. doi: 10.1371/journal.pone.0247498 (PMC7904228; doi:10.1371/journal.pone.0247498)
Supplement: S2 Table — (DOCX) [file pone.0247498.s004.docx]

S2 Table: Cohort 2 Patient Characteristics

| Variable | Level | N (%) = 40 |
| --- | --- | --- |
| Gender  Smoking  p16  Grade  T – Stage  Node Status  Stage | Male  Female  Never  Former  Current  Missing  Positive  Negative  Unknown  Missing  MD  NK  PD  WD  1  2  3  4  0  1  2  3  I  II  IV | 31 (77.5)  9 (22.5)  15 (38.5)  16 (41)  8 (20.5)  1  25 (67.6)  6 (16.2)  6 (15.0)  3  17 (42.5)  16 (40)  6 (15)  1 (2.5)  5 (12.5)  13 (32.5)  2 (5.0)  20 (50)  8 (20)  1 (2.5)  30 (75)  1 (2.5)  1 (2.5)  3 (7.5)  36 (90) |
