# Supplementary material for: Co-expression of fibroblast growth factor receptor 3 with mutant p53, and its association with worse outcome in oropharyngeal squamous cell carcinoma
Source: PLoS One. 2021 Feb 24;16(2):e0247498. doi: 10.1371/journal.pone.0247498 (PMC7904228; doi:10.1371/journal.pone.0247498)
Supplement: S3 Table — (DOCX) [file pone.0247498.s005.docx]

S3 Table. Expression Levels of FGFR3 and mp53 in Cohort 1

| Variable | Level | Expression level |
| --- | --- | --- |
| FGFR3  Mutant p53 (mp53)  Cytoplasmic mp53  Nuclear mp53 | Mean  Median  Minimum  Maximum  Std Dev  Missing  Mean  Median  Minimum  Maximum  Std Dev  Mean  Median  Minimum  Maximum  Std Dev  Mean  Median  Minimum  Maximum  Std Dev | 60.43  35.00  0.00  300.00  67.68  0.00  44.97  3.50  0.00  290.00  70.39  25.74  2.00  0.00  190.00  42.46  17.39  0.00  0.00  270.00  53.11 |
