# Supplementary material for: Co-expression of fibroblast growth factor receptor 3 with mutant p53, and its association with worse outcome in oropharyngeal squamous cell carcinoma
Source: PLoS One. 2021 Feb 24;16(2):e0247498. doi: 10.1371/journal.pone.0247498 (PMC7904228; doi:10.1371/journal.pone.0247498)
Supplement: S4 Table — (DOCX) [file pone.0247498.s006.docx]

S4 Table. Expression Levels of FGFR3 and mp53 in Cohort 2

| Variable | Level | N (%) = 40 |
| --- | --- | --- |
| FGFR3  Mutant p53 (mp53)  Cytoplasmic mp53  Nuclear mp53 | Mean  Median  Minimum  Maximum  Std Dev  Mean  Median  Minimum  Maximum  Std Dev  Mean  Median  Minimum  Maximum  Std Dev  Mean  Median  Minimum  Maximum  Std Dev | 100.57  100.00  0.00  280.00  74.15  82.42  63.33  0.00  240.00  70.36  78.65  60.00  0.00  285.00  81.07  21.62  0.00  0.00  210.00  51.27 |
