# Supplementary material for: Co-expression of fibroblast growth factor receptor 3 with mutant p53, and its association with worse outcome in oropharyngeal squamous cell carcinoma
Source: PLoS One. 2021 Feb 24;16(2):e0247498. doi: 10.1371/journal.pone.0247498 (PMC7904228; doi:10.1371/journal.pone.0247498)
Supplement: S5 Table — (DOCX) [file pone.0247498.s007.docx]

S5 Table. Correlation of FGFR3 with mP53 in Cohort 2.

|  | | FGFR3 | |
| --- | --- | --- | --- |
| Variable | N | Pearson CC | Pearson p-Value |
| Mutant p53 (mp53)  Cytoplasmic mp53  Nuclear mp53 | 35  32  32 | 0.287  -0.022  -0.195 | 0.095  0.904  0.285 |
