# Supplementary material for: Co-expression of fibroblast growth factor receptor 3 with mutant p53, and its association with worse outcome in oropharyngeal squamous cell carcinoma
Source: PLoS One. 2021 Feb 24;16(2):e0247498. doi: 10.1371/journal.pone.0247498 (PMC7904228; doi:10.1371/journal.pone.0247498)
Supplement: S7 Table — (DOCX) [file pone.0247498.s009.docx]

S7 Table. Cohort 2 Pearson Correlation Coefficient (p-values) between p16 and FGFR3, mp53, Cytoplasmic and Nuclear mp53

|  | mp53 | Cyto mp53 | Nuc mp53 | FGFR3 |
| --- | --- | --- | --- | --- |
| p16 | -0.42855  (<0.0001) | -0.37872  (<0.0001) | -0.25650  (0.0009) | -0.27633  (<0.0001) |
